# Supplementary material for: Are carbon emissions trading and green financial instruments synergistic? -Comprehensive quantitative research based on content analysis
Source: PLoS One. 2024 Mar 7;19(3):e0298601. doi: 10.1371/journal.pone.0298601 (PMC10919601; doi:10.1371/journal.pone.0298601)
Supplement: S1 Data — (ZIP) [file pone.0298601.s001.zip › Data/地级市Z_F工作报告.zip!/╦─┤¿/╦∞─■╩╨/└┤╫╘╣1⁄2╓┌║┼í╛┬φ┐╦╩2╛▌═°í┐_2014.docx]

2013年，在省委、省政府和市委的坚强领导下，在市人大、市政协的监督支持下，市政府团结和依靠全市人民，克服宏观经济下行的不利影响，沉着应对"6·30"特大洪灾的严峻挑战，全面实施"六大兴市计划"，全市经济社会发展取得长足进步。主要经济指标增速在全省排位靠前，呈现出稳中有进、稳中提质、稳中向好趋势。地区生产总值、规上工业增加值、社会消费品零售总额等10项主要经济指标增速进入全省前十位

　　2014年是在新起点上全面深化改革的开局之年，是全面实施"六大兴市计划"的重要一年，也是完成"十二五"规划目标的关键之年。今年政府工作的总体要求是：深入贯彻党的十八大、十八届二中、三中全会和省委十届四次全会精神，全面落实市委六届六次全会和市委经济工作会议部署，用改革统领经济社会发展全局，纵深推进"六大兴市计划"，促进全市经济社会持续健康发展

　　■首抓投资拉动，发展动能显著增强

　　◆项目支撑强劲有力 完成次级综合交通枢纽规划。遂资高速公路建成通车。遂广、遂西高速公路建设有序推进。中环线、观音湖隧道、机场迁建等重大项目顺利开工。大英、蓬溪到市城区快捷通道加快建设。

　　◆开放引资成效明显 成功引进投资亿元以上的项目103个，到位市外资金419亿元。成功举办"中国遂宁·东盟商贸投资洽谈会"。进出口获权企业达262家，进出口总额增长20%。

　　◆要素保障得到加强 报征土地1439公顷，创近三年新高；清理处置低效工业用地6115亩。桐子垭220KV和天星坝、射洪城西、金华110KV输变电工程按期建成投运，柳树电航工程和蓬溪220KV、金桥、仁里110KV输变电工程开工。

　　■重抓产业培育，发展质量明显提高

　　◆培优扶强成效显著 集中资源培育锂电、天然气、机械装备、电子信息四大主导产业。天齐锂业成功收购全球锂资源储量最大的澳大利亚泰利森公司，成功定向增发融资31亿元。大力培育现代物流、文化旅游、金融、总部经济四大服务业。

　　◆园区发展上档升级 成都置信参与开发的金桥新区完成投资18亿元，引进太平洋集团投资100亿元参与开发射洪锂电产业园。6个重点产业园区完成场平1.24万亩、拆迁82万平方米。国开区、物流港等四个园区被列入省政府"51025"重点产业园区培育名单。

　　◆现代农业实现突破 五个现代农业园区完成规划提升，园区建设完成投资31.9亿元。坚持规模化布局、集约化经营，新建柑橘基地5.9万亩、中药材基地2.3万亩，发展绿色蔬菜基地13万亩。深化现代畜牧业试点工作，规模以上畜牧企业达63家，畜牧业产值占农业总产值比重达43.8%。粮食总产达到156.4万吨，实现"七连增"。

　　■狠抓环境优化，发展保障更加坚实

　　◆城乡品位持续提升 世界荷花博览园、东西山城市森林公园等项目加快建设，城市主要进出口和主次干道风貌提升启动实施。"多城联创"扎实推进，我市成功通过国家卫生城市复评，成为国家智慧城市试点市。

　　◆政务效能大幅增强 建立"导办员"制度，设立"重大项目VIP室"，方便了群众和企业办事。开通"12345"政府服务热线，热线回应群众诉求的按期办结率达98.3 %。

　　◆生态环境更加优美 48户企业淘汰了落后产能，全市单位工业增加值能耗下降8.1%，单位GDP能耗下降4.2%。国控出川水质断面污染源综合整治项目完工，整治畜禽养殖企业85家，超额完成污染减排任务。

　　■抓实民生建设，发展成果惠及群众

　　◆民生工程深入人心 新建和改扩建公办幼儿园41所，新建中小学校舍15万平方米。基本药物制度覆盖所有公办医疗卫生机构。遂宁中心医院河东院区投入使用。全市食品药品监管体制改革基本完成。城镇登记失业率控制在4.17%以内，农村劳动力转移输出126.8万人次。

　　◆保障体系日渐完善 解决被征地农转非人员参加养老保险的历史遗留问题，新增参保人员12.9万人。城乡基本养老保险覆盖率达79%，城镇居民基本医疗保险参保率达98%，新农合参合率达98.7%。城乡低保月人均补助分别提高到197元和88元。

　　◆社会大局和谐稳定 大力开展"平安遂宁"创建，全市抢夺案件下降38.4%，抢劫案件下降19.2%。沉着应对"6·30"特大洪灾，夺取了抗洪救灾和灾后重建胜利。人口自然增长率控制在2.27‰以内。"六五"普法有序推进。

　　■深化改革攻坚，充分激活发展动能

　　◆加快经济领域改革 健全完善市场准入、财税扶持等政策。深化国有企业改革。加快财税体制改革。加快投融资体制改革。加快金融改革。

　　◆推动行政体制改革 突出简政放权。加强行政审批制度改革。建立权力清单制度。整合政务服务与公共资源交易平台建设。

　　◆统筹社会事业改革 加快教育领域综合改革。加快医药卫生体制改革。加快文化管理体制改革。抓好"单独两孩"政策实施。

　　■稳定投资扩大消费，保持经济健康发展

　　◆优化投向稳定投资 稳定政府性投资。增大产业投资比重。全力实施80个市级重大项目。

　　◆扩大有效消费需求 积极培育信息消费、健康养老等新的消费热点。

　　◆增强开放合作实效 加强招商队伍建设。深化专业招商、中介招商和以商招商。

　　■加快转方式调结构，推动产业转型升级

　　◆深入实施主导产业培育计划 坚持把培育四大主导产业和实施大企业扶持计划作为加快工业结构调整升级的重中之重。

　　◆积极培育服务业增长点 把现代物流业作为重中之重。加快金融服务中心建设；鼓励金融创新。

　　◆完善功能推动园区上档升级 深入实施园区"141工程"，将国开区打造成为全省外向型产业示范园区。

　　■强化"三农"工作，促进农业发展农民增收

　　◆提升现代农业发展效益 建立市级领导联系园区工作机制。加快生猪、果蔬等优质农业发展。

　　◆千方百计增加农民收入 推进农村产权制度改革。按照"风险可控、助农增收"的原则，探索成立农民投资理财增收平台。

　　◆建设一批幸福美丽新村 扎实抓好安居、蓬溪两个省级，船山、射洪、大英3个市级新农村示范县项目实施。

　　■坚持以人为核心，有序推进新型城镇化建设

　　◆优化城镇布局和发展形态 坚持基础设施和生态建设优先。开展"绿色城镇建设行动"。

　　◆提高城镇精细化管理水平 建立市向县（区）派驻规划督察员制度。深化智慧城市建设。

　　◆促进农业转移人口市民化 从衣食住行、社会保障等方面入手，解决好农业转移人口市民化过程中的困难和问题。

　　■顺应群众美好期待，持续保障和改善民生

　　◆突出办好民生实事 深入实施十大民生工程。

　　◆加强生态文明建设 深入推进林业"两大工程"，森林覆盖率提高0.6个百分点。

　　◆大力发展社会事业 深入推进义务教育学校标准化建设。做好第二代社会保障卡发放和应用工作。

　　◆依法加强社会治理 积极开展普法教育，加大法律援助。

　　政府自身建设

　　2014年，我们将加强政府自身改革建设，提升执行力和公信力，努力为群众提供更加优质高效的服务。

　　◆深入推进依法行政

　　◆强化执行力建设

　　◆推动工作作风转变

　　◆切实抓好廉政建设
